# Relationships Between Psychological Health and Academic Performance Among Undergraduate Students in the Third Year of the COVID-19 Pandemic: A Cross-Sectional Study

**DOI:** 10.3390/bs15091281

**Published:** 2025-09-18

**Authors:** Ram Lakhan, Maribel Vergara, Zoe Moore, Manoj Sharma

**Affiliations:** 1 Department of Health and Human Performance, Berea College, Berea, KY 40403, USA; mari.vergara280@gmail.com (M.V.); moorez@berea.edu (Z.M.); 2 Department of Social and Behavioral Health, School of Public Health, University of Nevada, Las Vegas, NV 89119, USA; manoj.sharma@unlv.edu; 3 Department of Internal Medicine, Kirk Kerkorian School of Medicine, University of Nevada, Las Vegas, NV 89154, USA

**Keywords:** psychological factors, depression, anxiety, well-being, sleep, academic performance, digital survey, public health

## Abstract

The COVID-19 pandemic increased mental health issues and heavily affected the academic performance of college students. The study aimed to assess the association of psychological health and behavioral factors with academic performance among undergraduate students during the third year of the COVID-19 pandemic. The study was conducted at a small liberal arts undergraduate college in rural Appalachia. A cross-sectional research design was followed. Data was collected online using Qualtrics in person in July 2021. Participants were selected randomly. World Health Organization-5, Perceived Stress Scale 4, The Pittsburgh Sleep Quality Index, Generalized Anxiety Disorder-7, and Patient Health Questionnaire scales for well-being, stress, sleep quality, anxiety, and depression were used, respectively. Spearman’s correlation, *t*-test, analysis of variance, and multiple regression were conducted. Overall well-being, perceived stress, generalized anxiety, depression, and sleep quality were found to be significantly different by gender and exercise. The Grade Point Average (GPA) was found to be negatively associated with depression and positively associated with Sleep quality. Findings suggest that students who have scored higher on the depression and sleep quality scale may be affected more during this COVID-19 pandemic in maintaining a good GPA. The findings of this study can help generate hypotheses for further research and guide interventions to address poor academic performance.

## 1. Introduction

Undergraduate students often experience stress related to academics, relationships, sexuality, work, time management, homesickness, and finances (Mofatteh, 2021). These factors can lead to emotional stress and affect their psychological and physical health (Elias et al., 2011; Karyotaki et al., 2020; Shankar & Park, 2016). Many students are not prepared enough to handle such adjustment issues. As a result, they are highly vulnerable to experiencing distress (Murphy et al., 2010), sleep problems (Forquer et al., 2008), anxiety, and even depression (Alansari, 2005; Rosenthal & Schreiner, 2000). Some of them may even engage in self-medicating negative behaviors, including smoking (Saravanan & Heidhy, 2014), overeating, and drinking (Bardone-Cone et al., 2012). Chronic stress, poor sleep, anxiety, and depression are potential factors that can affect overall academic performance and well-being and can be caused by smoking and drinking. These behaviors can also increase the risk for cancer, metabolic syndrome, and other chronic diseases (Johansen et al., 2010; Lee & Kowdley, 2012).

Numerous studies around the world have reported increased incidence of mental health issues and academic struggle from the beginning of the COVID-19 pandemic among students of all ages, academic disciplines, and educational levels, including undergraduates (Chernomas & Shapiro, 2013; Goebert et al., 2009; Sherina et al., 2004; Ramteke & Ansari, 2016; Syed et al., 2018). The sudden shift from in-person to online learning came with unimaginable challenges (Cortés-Albornoz et al., 2023). By the beginning of the third year of the COVID-19 pandemic, universities and colleges started resuming hybrid and in-person classes (Hine et al., 2025). This was the phase of readjusting to a semi or complete pre-COVID-19 learning atmosphere for many students (Hine et al., 2025). The increasing rates of vaccination, reduced mortality from COVID-19 were reducing fear in the overall population; however, fear and uncertainty were still present (Babicki et al., 2021). Poor mental health is associated with poor academic outcomes (Rožman et al., 2025; Zhang et al., 2024). However, it is also known that many students attempt to overcome their psychological problems on their own and do better academically and with their psychological health as time passes (Höhn et al., 2025). The COVID-19 pandemic was perceived as a shock, but as time passed, it can be assumed that people started resuming their pre-COVID-19 psychological health, which might have a favorable impact on the academic performance of the students (Höhn et al., 2025).

At this stage, it was a worthwhile idea to assess the situation of how undergraduate students at a liberal arts college were adjusting to their psychological health and how their psychological health and behaviors were associated with their academic performance. The research attempted to understand the overall profile of students on their perceived well-being, stress, sleep quality, anxiety, depression, tobacco use and smoking, alcohol consumption, exercise, and their association with self-reported academic performance. Madison County is a part of the Appalachia region where people in large numbers suffer from poverty, a higher unemployment rate, and lower household income (Wisnieski et al., 2021). Berea College provides completely free high-quality undergraduate education to students who fall below the poverty line. Berea College is a work college. In return for a completely free education, each student must work in the college for ten hours per week, and for that, they receive an hourly payment. Many students can save some money. Most students are the first in their families to attend college. Many students who attend Berea College either by themselves or with their families have experienced some form of vulnerability (Burnside, 1985). The association between psychological health and academic performance is well established in the literature (Rožman et al., 2025; Zhang et al., 2024). Although the COVID-19 situation was improving at this stage, fear and uncertainty were still present. Data was needed to develop a better return plan for the students to address their academic and psychological needs.

It is well-established that poor psychological health can impede on students’ ability to learn. Depression, anxiety, and stress can lead to poor attention and concentration, which can be a deterrent in learning, retaining information, and maintaining motivation (Deng et al., 2022; Grøtan et al., 2019). Poor academic performance and low motivation may lead to a student dropping out (Trusty et al., 2025). A prospective study found depression, anxiety, and stress were negatively associated with academic engagement and positively associated with dropout intentions among medical students (Sinval et al., 2025). Good psychological health can improve academic performance as it helps in managing stress effectively, being more socially connected, resilient, focused, and motivated. This study was planned to understand the overall psychological health of the students at this stage of the pandemic. The information obtained from this research can help identify gaps in existing student support systems and may also guide us to design more specific psychosocial plans in the hope of improving overall well-being and reducing the risk of drop-out that was still higher among the students, due to COVID-19.

## 2. Materials and Methods

The study utilized a cross-sectional research design. The research proposal was approved by the Berea College Institutional Review Board, KY, USA (Protocol #413). Primary author (RL) and her research assistants (MV and ZM) received stipend to conduct this research. Finances support was provided for material and other logistics under the Undergraduate Research and Creative Projects and Programs. Data was collected from undergraduate students at Berea College, Madison County, Kentucky, using a digital platform, Qualtrics, as well as in person. The informed consent form was also collected and provided digitally.

A survey form was created and piloted on 10 student volunteers. It included demographic, behavior-related questions, self-reported Grade Point Average (GPA), and standard scales for psychological health. The GPA is used as a numerical way of measuring academic performance. Psychological health includes stress, sleep quality, anxiety, depression, and overall well-being. The scales used were World Health Organization (WHO-5) also named as WHO (Five) Well-Being Index (1998 version) for overall well-being (World Health Organization, 2024), Perceived Stress Scale 4 (PSS-4) for stress (Cohen et al., 1983), The Pittsburg Sleep Quality Index (PSQI) for sleep quality (Buysse et al., 1989), Generalized Anxiety Disorder-7 (GAD-7) for anxiety (Löwe et al., 2008), and the Patient Health Questionnaire (PHQ-9) for Depression (Arroll et al., 2010).

### Statistical Analysis

A sample size of 194 for a cross-sectional, bivariate Pearson correlation, significant level (type I error = 0.05), power (type II error) = 0.80, and effect size (r) = 0.20 (medium effect) was found appropriate (Kohn & Senyak, 2024; Hulley, 2013). We planned to collect an additional 15% of data to fill the gap of inaccuracy and substantial missing information. We randomly selected four classes in each category (upcoming freshman (bridge program), rising sophomore, rising junior, and rising senior). They were given a digital survey through Qualtrics. College was also offering in-person classes during this semester, so one in-person class of each category was given a paper-and-pencil survey. This was performed to achieve the target sample. Incomplete forms were excluded from the analysis.

The characteristics of the participants and behavioral factors were described with means and standard deviations for metric variables and frequencies and percentages for categorical variables.

The *t*-test and analysis of variance (ANOVA) were used to examine the difference in all five psychological measures with gender, classification (year in undergraduate), ethnicity, country, and behavioral factors, including tobacco and smoking, drinking, and exercise. Spearman’s correlation was used for the strength and the direction of association between behavioral factors alcohol consumption, tobacco use, and exercise, with academic performance. Multiple regression analysis was conducted to assess the association of well-being, sleep quality, perceived stress, anxiety, and depression with self-reported GPA.

## 3. Results

Table 1 presents characteristics of the participants with the frequency for the sociodemographic factors, behavioral factors, and mean and standard deviation for self-reported GPA. The characteristics of the surveyed population were changing rapidly at this stage as students were returning to in-person classes. There is a chance that students’ behaviors with regards to exercise, smoking, and alcohol consumption might have changed to adjust to a new routine on campus from the routine at home when classes were taken online. Two hundred forty-four students (female 147 (60%), male 84 (34.4%), and other 6 (2.5%)) attending classes during the summer have participated in this research. The mean ± standard deviation of participants’ age was 20.22 ± 1.65 and GPA 3.49 ± 0.50. Of the participants, 13.1% classified themselves as emerging freshmen, 21.1% as rising sophomores, 27.9% as rising juniors, and 32.4% as rising seniors. Participants who identified as White totaled 50.8%, African American 16%, Hispanic or Latino 11.1%, Asian 4.9%, and other 9.8%. The origin of the country for 72.5% was the USA, while the other 19.7% were international students represented from several countries. As far as behavioral factors were concerned, 88.1% did not use tobacco, 57.8% did not consume alcohol, 49.2% exercised at least 150 min per week, 20.1% had some form of diagnosed disease or condition excluding mental illness, 86.5% were currently with COVID-19 vaccination, while 4.5% had no intention of having the vaccine. The previous semester (spring 2021) was taken online by 32.4%, online as well in person (hybrid form) by 48.4%, and fully in-person by 7%, while 1.6% took a leave of absence (Table 1).

In Table 2a, we have used ANOVA in assessing the difference in participants’ psychological health, including well-being, perceived stress, generalized anxiety, depression, and quality of sleep with their sociodemographic factors, including their gender, year of education (classification), ethnicity, country of origin, and mode of learning. Overall well-being, perceived stress, generalized anxiety, depression, and sleep quality were found to be significantly different by gender and exercise at least 150 min a week (*p* < 0.001). However, these measures remained unaffected by ethnicity, classification (the year of undergraduate program, freshmen, sophomore, etc.), mode of learning (online and in person), and national vs. international status (*p* > 0.05) (Table 2a).

In Table 2b, we have used a *t*-test to assess the difference in psychological health, including well-being, perceived stress, generalized anxiety, depression, and quality of sleep, with their behavioral factors, including tobacco use, alcohol consumption, and 150 min of exercise per week. The information on the association between sociodemographic and behavioral factors with psychological factors was important to understand the students’ overall profile and explore ideas to address issues. A difference in perceived stress was observed with tobacco use and generalized anxiety and depression with alcohol consumption (*p* < 0.001) (Table 2b).

In Table 3, Spearman’s correlation statistics were used to assess a difference in self-reported GPA with the behavioral factors, including tobacco use, alcohol consumption, and exercise for 150 min per week. The self-reported GPA was found to be lower who consumed alcohol (ρ = −1.84, *p*-value = 0.008); however, the GPA did not differ among those who used tobacco (ρ = −0.068, *p*-value = 0.326), and those who exercised at least 150 min per week (ρ = −0.021, *p*-value = 0.768) (Table 3).

Multiple regression analysis was conducted to evaluate the extent to which independent variables and psychological health were associated with the self-reported GPA of the students. The COVID-19 trauma included the death of someone from COVID-19 in the family or an extremely painful self-experience that included hospitalization for the treatment was controlled in the analysis. The regression model was found significant in the analysis of variance (ANOVA) (F = 3.353, *p*-value = 0.006). The R^2^ = 0.057 indicated a 5.7% variability in students’ GPA explained by variance in psychological factors. The following regression equation has emerged from the model. Regression equation—GPA = 3.89 + −0.26 (depression) + 0.015 (sleep quality). Self-reported GPA was found to be lower by 0.26% among participants who had scored one point higher for depression, while self-reported GPA was found to be 0.15% higher among the participants who scored one point higher in sleep quality (Table 4).

## 4. Discussion

This study aimed to assess the association of psychological factors with self-reported GPAs of undergraduate students when the vaccine became available, and people started resuming their pre-COVID-19 schedule. Students were depressed and consumed alcohol, they reported poor GPA. The self-reported GPA was better among those who had better sleep quality. These findings matched with a recently published study (Gavurova et al., 2020). Researchers have found a very high rate of depressive symptoms (63.4%) and anxiety (mild 70.8%, moderate 29.2%) among students, even in the post-COVID-19 phase, when a large population was vaccinated and resumed in-person functioning around the world (Gottlieb et al., 2024). To get an idea of how people are adjusting to their mental health concerns, a study was conducted in the Appalachia region at the beginning of 2021 that attempted to assess the rate of depressive symptoms and anxiety for pre-, during, and phasing out the COVID-19 pandemic showed resilience and declining of depressive and anxiety symptoms as the time passed and people started resuming to pre-COVID-19 routines (Lakhan et al., 2021). In the same context, we found a systematic review and meta-analysis of 15 countries that reported a very high rate of anxiety (39%), depression (31%), stress (26%), post-traumatic stress disorder, and poor sleep quality (50.5%) in university students (Batra et al., 2021). A meta-analysis of 39 studies from 19 countries on academic achievement found students struggling to catch up with their learning (Di Pietro, 2023).

An association of alcohol consumption with self-reported poor GPA and differences in depression and generalized anxiety may be an indication of self-medication behavior among struggling students. Students might have started alcohol consumption to cope and adjust themselves. There is a possibility that these students might already be performing poorly academically, which is exacerbated further due to the pandemic. An increase in alcohol consumption and its association with poor academic performance was also found in other studies (Charles et al., 2021; Jodczyk et al., 2022; Vargas-Ramos et al., 2021).

Perceived stress was observed differently between tobacco and non-tobacco users in this study. According to another study conducted at a Polish university around the same time, it found an increased tendency of tobacco and alcohol consumption among students as a way of coping. However, they did not find this behavior of increased tobacco consumption turning into a habit (Jodczyk et al., 2022).

Students with good sleep quality were found to be doing better with their GPA. A positive association between good sleep quality with GPA is already established in other studies (Musshafen et al., 2021). Sleep is a natural behavior that helps to restore energy and improve mental well-being, and reduce stress (Vandekerckhove & Wang, 2018). It is probable that fewer opportunities for outdoor activities during this time may have also helped some participants to get more sleep to relax and thus overcome their psychological stress. A study conducted in the USA with college students, just predating this research, found that reduced opportunities for outdoor activities for college students played a favorable role for many students to use that time to get more sleep (Vandekerckhove & Wang, 2018). Data in this study were collected from the students who were taking courses during the summer semester. Usually, students who take classes in the summer often have different priorities and motivations. Additionally, the academic workload during the summer semester is relatively lower. There is a chance that many students found more time to relax and sleep, which had an impact on academic performance (Kowalsky et al., 2023; Vandekerckhove & Wang, 2018). We are also aware that psychological health in all areas of psychological factors was found to be favorably associated with 150 min of exercise per week.

The favorable effect of exercise on psychological well-being and overall mental health has been observed in several studies. Cross-sectional studies conducted around the same time of this research found exercising behavior to be a protective factor against depression and psychological stress among university students. Good GPA was also found to be favorably associated with regular exercise; however, studies cannot establish a causal correlation between good GPA and regular exercise based on the cross-sectional nature of research (Xiang et al., 2020; Gosadi, 2024).

All participants included in this study come from a very poor socioeconomic background. Their motivation for education may be different from the larger student population in the USA; therefore, these findings must be read carefully, while other studies showed a short-term association with tobacco and alcohol consumption, but did not lead to addiction. In the absence of comparison, it is difficult to say that alcohol consumption and tobacco use will not increase in this population. The risk of dropping out of education cannot be denied for this population, considering the findings of another study in which depression, anxiety, and stress were found to be positively associated with the intention to drop out (Xiang et al., 2020). Participants in this study with poorer psychological health also suffer from lower GPA, which puts a dual psychological burden and risk of dropping out. The institute did not mandate attendance for in-person as well as online classes to offer more flexibility, dining hours were extended, and gym equipment was spread out in a larger building to avoid a risk of congregation. The academic load was relatively lower during the summer semester when data were collected, so there is a possibility that the association between psychological health with behavioral factors may change when a larger number of students later resume in person.

### Strengths and Limitations

The study has provided some information about academic performance in association with psychological and behavioral factors among undergraduate students at a liberal arts college in Appalachia. The findings at that time were very relevant in the lack of published research. However, more research was conducted around that time and published later, which showed a similar pattern, but this population, being unique in terms of socioeconomic position and mostly being first-generation in college, presents important information that can be applied to a similar population. Self-reported GPAs may have some bias. Data on current academic load, social support available at the college, completely free education, and mandatory ten hours weekly paid work were not collected. These factors might have some influence on the findings. Being a cross-sectional research design, it does not establish a causative relationship. As a strength, this study helps to generate hypotheses for longitudinal and experimental research designs.

## 5. Conclusions

A few findings of this research were consistent with the studies conducted globally at that time with university students. However, this study indicates a stronger need for further research with a robust methodology with a larger sample of students including those who have taken a long leave of absence, dropped out completely from the college, indicated intention to return to campus or staying online, and who said they need more time to decide for the purpose of better understanding their level of resilience, motivation for returning to campus, continuing their education, and their rationales for consuming alcohol and tobacco. It is also important to get a deeper understanding of the motivation for regular exercise and its connection with the GPA. If the majority of participants are engaged in regular exercise to cope with stress, a more tailored program for promoting physical activities along with mental health support could be developed.

## Figures and Tables

**Table 1:** Characteristics of participants with behavioral factors (N = 244).

Demographic Characteristics	N/Categories	Mean	SD
Age	239	20.22	1.65
GPA (Self-reported)	211	3.499	0.505
Gender		N	%
	Male	84	34.4
	Female	147	60.2
	Others	6	2.5
Ethnicity	White	124	50.8
	African American	39	16.0
	Hispanic or Latino	27	11.1
	Asian	12	4.9
	Other	24	9.8
Educational Classification	Freshmen	32	13.1
	Sophomores	52	21.3
	Juniors	68	27.9
	Seniors	79	32.4
Country of Origin	USA	177	72.5
	International	48	19.7
Behavioral Factors	Yes/No	N	%
Use tobacco	No	215	88.1
	Yes	10	4.1
Consume alcohol	No	141	57.8
	Yes	84	34.4
Exercise 150 min per week	No	102	41.8
	Yes	120	49.2
Have a disease or condition	No	175	71.7
	Yes	49	20.1
COVID-19 vaccination	No/also not planning	11	4.5
	Fully vaccinated	211	86.5
Education mode in Spring 2021	In-person	17	7.0
	Online	79	32.4
	Hybrid	118	48.4
	Leave	4	1.6

**Table 2:** a. The association between participants’ psychological and socio-demographic factors (n-244). b. The association between participants’ psychological and behavioral factors (n-244).

(a)						
Sociodemographic Categories	Psychological Factors	Categories	Mean	Sd	ANOVA	
					F	*p*-Value
Gender	Well-being	Females	11.92	5.037	7.210	0.001
		Males	14.10	5.679		
		Others	6.25	5.123		
	Perceived stress	Females	8.15	3.173	9.602	0.001
		Males	6.41	3.466		
		Others	11.25	2.630		
	Generalized anxiety	Females	10.79	5.769	11.314	0.001
		Males	7.96	5.896		
		Others	19.50	3.000		
	Depression	Females	11.48	7.090	7.251	0.001
		Males	8.91	6.610		
		Others	20.25	5.620		
	Quality sleep	Females	13.74	7.022	7.507	0.001
		Males	11.25	6.681		
		Others	23.00	3.559		
Classification	Well-being	Freshmen	13.46	4.451	0.320	0.811
		Sophomores	12.58	5.538		
		Juniors	12.33	5.637		
		Seniors	12.41	5.451		
	Perceived stress	Freshmen	7.57	2.962	0.111	0.954
		Sophomores	7.47	3.355		
		Juniors	7.79	3.446		
		Seniors	7.50	3.631		
	Generalized anxiety	Freshmen	9.65	6.079	0.686	0.561
		Sophomores	9.96	6.197		
		Juniors	10.76	6.303		
		Seniors	9.29	5.673		
	Depression	Freshmen	10.88	7.560	0.763	0.516
		Sophomores	10.39	7.265		
		Juniors	11.85	7.384		
		Seniors	10.08	6.604		
	Quality sleep	Freshmen	13.54	9.060	0.126	0.945
		Sophomores	12.54	6.246		
		Juniors	13.20	6.921		
		Seniors	12.99	7.016		
Ethnicity	Well-being	African Americans	13.24	5.465	1.079	0.368
		Whites	12.13	5.528		
		Asians	14.67	4.960		
		Hispanics/Latinos	13.44	5.139		
		Others	11.77	5.433		
	Perceived stress	African Americans	8.34	3.505	0.820	0.514
		Whites	7.51	3.659		
		Asians	6.67	2.902		
		Hispanics/Latinos	7.12	2.372		
		Others	7.64	3.170		
	Generalized anxiety	African Americans	9.76	5.479	1.077	0.369
		Whites	10.62	5.960		
		Asians	9.09	6.204		
		Hispanics/Latinos	8.00	5.979		
		Others	9.68	7.260		
	Depression	African Americans	11.11	8.037	0.813	0.518
		Whites	11.28	6.993		
		Asians	9.33	7.365		
		Hispanics/Latinos	8.71	5.820		
		Others	10.36	7.148		
	Quality sleep	African Americans	11.50	8.027	1.479	0.210
		Whites	13.37	7.059		
		Asians	11.08	6.022		
		Hispanics/Latinos	12.46	5.233		
		Others	15.70	7.406		
Country	Well-being	USA	12.33	5.526	1.988	0.160
		International	13.58	5.069		
	Perceived stress	USA	7.71	3.454	1.245	0.266
		International	7.08	3.254		
	Generalized anxiety	USA	10.22	5.963	2.151	0.144
		International	8.73	6.344		
	Depression	USA	11.15	7.162	3.045	0.082
		International	9.07	6.780		
	Quality sleep	USA	13.37	7.228	2.177	0.142
		International	11.62	6.354		
Mode of Learning	Well-being	In-person	14.31	5.558	1.375	0.251
		Online	12.13	4.916		
		Hybrid	12.83	5.690		
		Leave	9.00	7.439		
	Perceived stress	In-person	7.69	2.750	0.184	0.907
		Online	7.53	3.343		
		Hybrid	7.48	3.595		
		Leave	8.75	4.031		
	Generalized anxiety	In-person	11.07	6.595	0.618	0.604
		Online	9.78	5.410		
		Hybrid	9.76	6.429		
		Leave	13.25	6.500		
	Depression	In-person	11.44	6.966	1.208	0.308
		Online	9.95	5.808		
		Hybrid	10.96	7.844		
		Leave	16.25	8.261		
	Quality sleep	In-person	13.63	8.740	0.087	0.967
		Online	13.07	6.692		
		Hybrid	13.01	7.049		
		Leave	14.50	9.574		
(b)						
Behavioral Factors	Psychological Factors	Beh. Factors (no/yes)	Mean	Sd	t-value	*p*-value
Tobacco user	Well-being	No	12.78	5.372	1.88	0.061
		Yes	9.33	5.723		
	Perceived stress	No	7.46	3.375	−2.10	0.036
		Yes	9.89	3.621		
	Generalized anxiety	No	9.76	6.093	−1.736	0.084
		Yes	13.33	4.555		
	Depression	No	10.48	7.075	−1.650	0.100
		Yes	14.44	6.267		
	Quality sleep	No	12.76	7.020	−1.681	0.094
		Yes	16.78	6.685		
Alcohol consumption	Well-being	No	13.04	5.447	1.529	0.128
		Yes	11.88	5.353		
	Perceived stress	No	7.33	3.419	−1.372	0.171
		Yes	7.99	3.361		
	Generalized anxiety	No	9.25	6.125	−2.197	0.029
		Yes	11.14	5.791		
	Depression	No	9.87	7.093	−2.312	0.022
		Yes	12.20	6.913		
	Quality sleep	No	12.45	7.322	−1.478	0.141
		Yes	13.95	6.550		
Exercise	Well-being	No	11.40	5.307	−3.365	0.001
		Yes	13.81	5.199		
	Perceived stress	No	8.16	3.371	2.643	0.009
		Yes	6.96	3.286		
	Generalized anxiety	No	10.90	6.073	2.385	0.018
		Yes	8.93	5.843		
	Depression	No	11.94	7.047	2.633	0.009
		Yes	9.41	6.810		
	Quality sleep	No	14.30	7.253	2.517	0.013
		Yes	11.84	6.797		

**Table 3:** Association of GPA with behavioral factors.

Behavioral Factors	Mean (SD)	N	ρ	*p*-Value
GPA	3.499 (0.50)	211		
Uses tobacco		210	−0.068	0.326
Consumes alcohol		209	−0.184	0.008
Exercise 150 m/week		207	−0.021	0.768

**Table 4:** Association of GPA with psychological factors.

Model Summary					
R	R^2^	Adj R^2^	Std. Error	ANOVA	
0.286	0.082	0.057	0.4951	F	*p*-value
				3.353	0.006
Regression					
Psychological factors	B	Std. Error	95% CI		*p*-value
			Lower	Upper	
Constant	3.890	0.244	3.408	4.372	<0.001
Well-being	−0.012	0.011	−0.033	0.009	0.246
Perceived Stress	−0.016	0.016	−0.048	0.016	0.328
Generalized anxiety	−0.003	0.010	−0.024	0.017	0.746
Depression	−0.026	0.010	−0.045	−0.007	0.007
Sleep Quality	0.015	0.007	0.001	0.029	0.042

## Data Availability

Data are available with the primary author (R.L.). Interested scholars may request data for scholarly purposes. Genuine requests will be accepted favorably. However, the primary author (R.L.) reserves the right to deny the request.
